# Autistic traits, systemising, empathising, and theory of mind in transgender and non-binary adults

**DOI:** 10.1186/s13229-020-00378-7

**Published:** 2020-09-29

**Authors:** Karson T. F. Kung

**Affiliations:** 1 Department of Psychology, University of Hong Kong, Pokfulam, Hong Kong; 2 Gender Development Research Centre, Department of Psychology, University of Cambridge, Cambridge, UK; 3 School of Psychology, University of Kent, Kent, UK

**Keywords:** Autism, Empathy, Extreme male brain, Gender minority, Non-binary, Systemising, Theory of mind, Transgender

## Abstract

**Background:**

Prior research examining autistic traits in gender minority adults has reported mixed findings. Most prior studies did not include non-binary individuals. Little is known about the mechanisms shaping autistic traits in gender minority adults. This study examined autistic traits, as well as constructs related to the extreme male brain theory of autism and the mindblindness theory, in transgender and non-binary adults.

**Methods:**

An online survey was conducted to assess autism-related traits in 323 gender minority adults, including 74 transgender men (individuals assigned female at birth and identify as a man), 95 transgender women (individuals assigned male at birth and identify as a woman), 104 non-binary AFAB (individuals assigned female at birth and identify as non-binary), and 50 non-binary AMAB (individuals assigned male at birth and identify as non-binary). Autistic traits, systemising, empathising, and Theory of Mind (ToM) were measured using the Autism Spectrum Quotient (AQ), the short forms of the Systemising Quotient (SQ-Short) and the Empathy Quotient (EQ-Short), the 10-item version of the Empathy Quotient (EQ-10) and the Reading the Mind in the Eyes Test (Eyes Test). Participants’ scores on these measures were compared with previously published scores based on large-scale general population samples including thousands of participants.

**Results:**

On average, compared with control females in the general population samples, both transgender men and non-binary AFAB scored significantly higher on the AQ and the SQ-Short but scored significantly lower on the EQ-Short, the EQ-10, and the Eyes Test. No clear or consistent group differences emerged when transgender women and non-binary AMAB were compared with control males.

**Limitations:**

The present study does not have a large sample of gender minority adults. It has been argued that the measures employed may not provide a precise assessment of the psychological constructs of interest. The present study has a “non-clinical” sample. However, not all gender minorities have access to or require clinical services, and so a “non-clinical” sample may be more representative of the gender minority community as a whole than samples recruited through clinics.

**Conclusions:**

The current findings suggest a “masculinised” autism-related profile and reduced ToM in transgender men and in non-binary AFAB. These findings might be interpreted to support the extreme male brain theory of autism and the mindblindness theory. Further research is needed to corroborate these findings.

## Background

Gender minority is a term that can be used to describe individuals whose gender identity does not match their sex assigned at birth. Several studies have compared average group differences in autistic traits between gender minority adults and control adults. Jones et al. [1] conducted the first study on this topic and found elevated autistic traits in gender minority adults assigned female at birth compared with control females [1]. Similar findings have been reported in subsequent studies [2–5, but also see 6]. Regarding comparisons between gender minority adults assigned male at birth and control males, findings from these prior studies were mixed [1–6]. All these studies employed versions of the Autism Spectrum Quotient, a self-reported questionnaire, to assess autistic traits [1–6]. Prior studies recruited gender minority adults through clinics [1, 2, 4–6] and online platforms [3]. All prior studies included transgender men and transgender women [1–6], and two of the studies also included non-binary individuals [2, 3]. Only one study analysed data for non-binary individuals separately, although results were generally similar across the transgender and non-binary groups [3]. In addition to the studies examining autistic traits in gender minority adults, other lines of research have reported increased cross-gender identification, or gender variance, in autistic individuals [7, 8] and a higher prevalence of autism in individuals with gender dysphoria [9, 10]. A recent large-scale study has reported higher rates of autism and other neurodevelopmental and psychiatric conditions in gender minority individuals [11] .

Little is known about the mechanisms underlying the elevation in autistic traits observed in gender minority adults assigned female at birth in prior research [1–5]. A popular theoretical framework used in prior research [1–6] is the extreme male brain theory of autism (EMB) [12], although the theory does not specifically focus on gender minorities. According to the EMB, autism can be considered as an extreme of a “male brain” characterised by increased systemising and decreased empathising [12]. A hypothesis related to the EMB proposes that heightened early androgen exposure contributes to the development of an ‘extreme male brain’ [13]. There is some empirical support for the links between early androgen exposure and systemising, empathising, and autistic traits, although findings have not been consistent across studies [14–22]. There is also evidence supporting an association between heightened early androgen exposure and reduced female-typical gender identity [23, 24]. Another relevant theoretical framework is the mindblindness theory, which proposes that in autism Theory of Mind (ToM), sometimes referred to as mind-reading, is impaired [25]. Although the mindblindness theory does not focus on gender differences or gender identity, males in the general population tend to score lower on certain measures of ToM than females [26, 27]. It has been proposed that heightened early androgen exposure may reduce ToM [18, 28]. Therefore, it is possible that the elevated autistic traits in gender minority adults assigned female at birth are driven by increased systemising and reduced empathising and ToM.

There is limited research assessing the relevant theoretical constructs in gender minorities. Di Ceglie et al. [29] conducted the first study on systemising and empathising, using parent-reported questionnaires in a clinic-based sample of gender minority adolescents. This study found lower empathising and unaltered systemising in adolescent transgender boys compared with control females, whilst there were no differences in empathising or systemising between adolescent transgender girls and control males [29]. More recently, a study based on an online sample of transgender, non-binary, and control adults reported mixed findings regarding self-reported empathising and systemising, as well as ToM assessed by the Reading the Mind in the Eyes Test [3]. These mixed findings may be attributable to the small samples in the studies; there were 35 gender minority adolescents in total across two subgroups in the first study [29] and 109 gender minority adults in total across four subgroups in the second study [3].

The present study extends prior research by comparing gender minority adults’ scores on measures of autistic traits, systemising, empathising, and ToM to previously published scores on the same measures from large-scale general population studies including thousands of participants. Based on prior research on autistic traits in gender minority adults [1–5], as well as the EMB and the mindblindness theory [12, 25], it was hypothesised that gender minority adults assigned female at birth would show increased autistic traits and systemising but reduced empathising and ToM compared with control females. Since prior findings regarding autistic traits in gender minority adults assigned male at birth were mixed, there were no specific hypotheses for comparisons between gender minority adults assigned male at birth and control males.

## Methods

### Participants

In 2017, gender minority adults were recruited via numerous charities, organisations, and support groups specialising in sexual and gender minority issues in the UK and the US. These groups were approached via email and were asked to circulate an online survey invitation through their mailing lists and social media platforms. Participants completed the online survey on the Qualtrics platform. The survey was approximately 20 minutes long. In the survey, participants were asked to provide information about their “Sex Assigned At Birth” and “Gender Identity”. For sex assigned at birth, they were given three options: “Male”, “Female”, and “Other”. For gender identity, they were given four options: “Man”, “Woman”, “Non-binary”, and “Other”. There were 323 gender minority adults, including 74 transgender men (individuals assigned female at birth and identify as a man; mean age = 32.88, SD = 14.59), 95 transgender women (individuals assigned male at birth and identify as a woman; mean age = 42.14, SD = 17.02), 104 non-binary AFAB (individuals assigned female at birth and identify as non-binary; mean age = 28.77, SD = 11.79), and 50 non-binary AMAB (individuals assigned male at birth and identify as non-binary; mean age = 42.92, SD = 14.34). The overall sample (196 from the UK, 127 from the US) had a mean age of 35.83 (SD = 15.69; range = 18–76). Two participants choosing “Other” for the sex assigned at birth question and 10 cisgender participants were excluded from the current study, because the samples in these groups were too small for statistical analyses.

### Measures

Autistic traits, systemising, and empathising were assessed by self-reported questionnaires including the Autism Spectrum Quotient (AQ; 50-item) [30] and the short forms of the Systemising Quotient (SQ-Short; 25-item) and the Empathy Quotient (EQ-Short; 22-item) [31]. A 10-item version of the EQ (EQ-10) [32] can also be derived using items from the EQ-Short. ToM was assessed using the revised version of the Reading the Mind in the Eyes Test (Eyes Test) [33], which consists of 36 grey-scale items/photos of people focusing on the area of the eyes. Participants were asked to identify the mental state of the person in each item/photo. Details of these measures, including their scoring methods and psychometric properties, can be found in the validation studies [30–33]. As indicated by Cronbach’s *α*, these measures had good internal consistency in the current study (AQ: *α* = 0.87; SQ-Short: *α* = 0.87; EQ-Short: *α* = 0.93; EQ-10: *α* = 0.86; Eyes Test: *α* = 0.63).

### General population samples as control groups

The current sample’s scores on these measures were compared with previously published scores on the same measures from general population samples of adults in large-scale studies. Descriptive statistics were extracted from the published studies for statistical comparisons. Descriptive statistics of the current sample and the general population samples are summarised in Table 1. For the AQ, control samples included 2562 females and 1344 males from Baron-Cohen et al. [34] and 298,084 females and 152,310 males from Ruzich et al. [35]. Other control comparisons were based on 1038 females and 723 males from Wakabayashi et al. [31] for the SQ-Short and the EQ-Short, 412,062 females and 259,544 males from Greenberg et al. [32] for the EQ-10, and 44,574 females and 43,482 males from Warrier et al. [36] for the Eyes Test. Participants in these general population studies were between 16 and 89 years of age.

**Table 1:** Sample sizes, measures, and descriptive statistics for the current study and the general population studies

	AQ		SQ-Short		EQ-Short		EQ-10		Eyes Test	
	*n*	Mean (SD)	*n*	Mean (SD)	*n*	Mean (SD)	*n*	Mean (SD)	*n*	Mean (SD)
Current study										
Transgender men	72	22.98 (9.02)	72	20.83 (9.45)	72	20.11 (10.90)	72	8.38 (5.21)	74	26.57 (4.80)
Non-binary AFAB	98	26.55 (8.38)	97	21.28 (9.81)	97	19.50 (10.71)	97	8.33 (4.98)	104	26.73 (4.20)
Transgender women	90	20.37 (7.76)	89	23.64 (10.01)	89	23.96 (9.54)	89	10.50 (4.80)	95	26.25 (3.58)
Non-binary AMAB	48	22.00 (7.05)	47	22.85 (9.75)	47	23.21 (9.42)	47	10.30 (4.31)	50	26.95 (4.22)
Baron-Cohen et al. [34]										
Females	2562	17.1 (7.6)		–	–	–	–	–	–	–
Males	1344	20.3 (7.8)	–	–	–	–	–	–	–	–
Ruzich et al. [35]										
Females	298,084	18.95 (8.52)	–	–	–	–	–	–	–	–
Males	152,310	21.55 (8.82)	–	–	–	–	–	–	–	–
Wakabayashi et al. [31]										
Females	–	–	1038	15.4 (8.77)	1038	26.0 (8.27)	–	–	–	–
Males	–	–	723	24.1 (9.55)	723	20.7 (8.46)	–	–	–	–
Greenberg et al. [32]										
Females	–	–	–	–	–	–	412,062	10.79 (4.84)	–	–
Males	–	–	–	–	–	–	259,544	8.87 (4.75)	–	–
Warrier et al. [36]										
Females	–	–	–	–	–	–	–	–	44,574	27.85 (3.55)
Males	–	–	–	–	–	–	–	–	43,482	27.08 (3.75)

AFAB = individuals assigned female at birth; AMAB = individuals assigned male at birth. The variation in decimal places reported in the table reflects differing report styles across published studies

### Analytical approach

Comparisons between the current sample and the control groups were made according to the participants’ sex assigned at birth (i.e., gender minority adults assigned female at birth were compared to control females and gender minority adults assigned male at birth were compared to control males). Within-sex analyses were conducted, because prior studies on this topic tend to focus on comparisons based on sex assigned at birth and because the relevant theoretical frameworks are generally based on genetic sex or sex assigned at birth rather than gender identity. Within each sex assigned at birth, transgender and non-binary individuals were separately compared to the control samples, because there is little research on non-binary individuals and separate analyses may usefully explore and detect potential group differences. One-sample *t*-tests were conducted to evaluate the statistical significance of the average group differences. All tests were two-tailed with alpha set at 0.05. The Cohen’s *d* statistic was used as an effect size indicator to evaluate the magnitude of the average group differences (small effect: *d* = 0.2; medium effect: *d* = 0.5; large effect: *d* = 0.8) [37].

## Results

### Transgender men and non-binary AFAB versus females in general population samples

The same results pattern was found in transgender men and in non-binary AFAB. Compared with control females in the general population samples, both transgender men and non-binary AFAB scored significantly higher on the AQ and the SQ-Short, but scored significantly lower on the EQ-Short, the EQ-10, and the Eyes Test. Details of inferential statistics and effect sizes are provided in Table 2.

### Transgender women and non-binary AMAB versus males in general population samples

There were no significant differences in AQ scores between either transgender women or non-binary AMAB and control males in the general population samples. Regarding SQ-Short scores, neither transgender women nor non-binary AMAB differed significantly from control males in the general population study. Compared with control males, transgender women scored significantly higher on the EQ-Short, the EQ-10, but scored significantly lower on the Eyes Test. Compared with control males, non-binary AMAB scored marginally significantly higher on the EQ-Short and significantly higher on the EQ-10, but did not differ significantly in terms of Eyes Test scores. Details of inferential statistics and effect sizes are provided in Table 2.

**Table 2:** Inferential statistics and effect sizes for the group comparisons

Comparison	Measure and control study	Direction of Effect	*t*	*p*	Cohen’s *d*	95% CI for *d*
Transgender men vs. control females	AQ (vs. Baron-Cohen et al.) [34]	Transgender men > control females	5.54	< 0.001	0.77	[0.53, 1.00]
	AQ (vs. Ruzich et al.) [35]	Transgender men > control females	3.80	< 0.001	0.47	[0.24, 0.70]
	SQ-Short [31]	Transgender men > control females	4.88	< 0.001	0.62	[0.38, 0.86]
	EQ-Short [31]	Transgender men < control females	− 4.58	< 0.001	− 0.70	[− 0.94, − 0.46]
	EQ-10 [32]	Transgender men < control females	− 3.93	< 0.001	− 0.50	[− 0.73, − 0.25]
	Eyes Test [36]	Transgender men < control females	− 2.29	0.025	− 0.36	[− 0.59, − 0.13]
Non-binary AFAB vs. control females	AQ (vs. Baron-Cohen et al.) [34]	Non-binary AFAB > control females	11.16	< 0.001	1.24	[1.03, 1.44]
	AQ ( vs. Ruzich et al.) [35]	Non-binary AFAB > control females	8.98	< 0.001	0.89	[0.69, 1.09]
	SQ-Short [31]	Non-binary AFAB > control females	5.90	< 0.001	0.66	[0.45, 0.87]
	EQ-Short [31]	Non-binary AFAB < control females	− 5.98	< 0.001	− 0.76	[− 0.97, − 0.55]
	EQ-10 [32]	Non-binary AFAB < control females	− 4.86	< 0.001	− 0.51	[− 0.71, − 0.31]
	Eyes Test [36]	Non-binary AFAB < control females	− 2.72	0.008	− 0.32	[− 0.51, − 0.12]
Transgender women vs. control males	AQ (vs. Baron-Cohen et al.) [34]	Transgender women > control males	0.84	0.993	0.01	[− 0.20, 0.22]
	AQ (vs. Ruzich et al.) [35]	Transgender women < control males	− 1.44	0.152	− 0.13	[− 0.34, 0.07]
	SQ-Short [31]	Transgender women < control males	− 0.43	0.666	− 0.05	[− 0.27, 0.17]
	EQ-Short [31]	Transgender women > control males	3.22	0.002	0.38	[0.16, 0.60]
	EQ-10 [32]	Transgender women > control males	3.21	0.002	0.34	[0.14, 0.55]
	Eyes Test [36]	Transgender women < control males	− 2.27	0.026	− 0.22	[− 0.42, − 0.02]
Non-binary AMAB vs. control males	AQ (vs. Baron-Cohen et al.) [34]	Non-binary AMAB > control males	1.67	0.102	0.22	[− 0.07, 0.51]
	AQ (vs. Ruzich et al.) [35]	Non-binary AMAB > control males	0.44	0.661	0.05	[− 0.23, 0.33]
	SQ-Short [31]	Non-binary AMAB < control males	− 0.88	0.384	− 0.13	[− 0.43, 0.16]
	EQ-Short [31]	Non-binary AMAB > control males	1.83	0.074	0.29	[− 0.00, 0.59]
	EQ-10 [32]	Non-binary AMAB > control males	2.27	0.028	0.30	[0.02, 0.59]
	Eyes Test [36]	Non-binary AMAB < control males	− 0.21	0.832	− 0.03	[− 0.31, 0.24]

AFAB = individuals assigned female at birth; AMAB = individuals assigned male at birth

### Age and geographical location

In the present study, there were no significant effects of age or geographical location on any of the measures in any of the subgroups or in the overall sample. Also, large-scale studies examining the effects of age and geographical location on the employed measures have reported negligible effects [32, 35, 36]. Hence, the current findings may not be attributable to any potential differences in age or location between the current and control samples.

### High-scoring individuals

A cut-off score of 35+ on the AQ has been used to categorise individuals as having the narrow autism phenotype [1, 38]. 15% of transgender men, 19% of non-binary AFAB, 3% of transgender women, and 2% of non-binary AMAB met the cut-off. Statistical comparisons cannot be made using the large-scale general population studies [34, 35], because those studies focused on average scores and did not examine the proportion of high-scoring individuals.

### “Brain types”

Based on differences between standardised SQ-Short and EQ-Short scores, participants can be classed into one of five cognitive profiles, or “brain types”. The relevant calculations and “brain type” classification in the current study were performed following the procedures outlined in Wakabayashi et al. [31]. Descriptive statistics of “brain types” are summarised in Table 3. Approximately half of the cells in the current sample had a low value (*n* < 10), and thus no statistical tests were conducted to compare the distribution of “brain types” between subgroups in the current study and the general population sample [31]. However, descriptive statistics seem to indicate that, compared with control females, both transgender men and non-binary AFAB were more likely to have “brain types” of high systemising and low empathising. By contrast, it seems that the distribution of “brain types” was similar amongst transgender women, non-binary AMAB, and control males.

**Table 3:** Descriptive statistics of “brain types” in the current sample and in Wakabayashi et al. [31]

	Extreme Type E	Type E	Type B	Type S	Extreme Type S
Transgender men	6.9% (*n* = 5)	11.1% (*n* = 8)	43.1% (*n* = 31)	16.7% (*n* = 12)	22.2% (*n* = 16)
Non-binary AFAB	3.1% (*n* = 3)	7.2% (*n* = 7)	49.5% (*n* = 48)	16.5% (*n* = 16)	23.7% (*n* = 23)
Control females [31]	15.4% (*n* = 160)	25.9% (*n* = 269)	46.6% (*n* = 484)	8.5% (*n* = 88)	3.6% (*n* = 37)
Transgender women	4.5% (*n* = 4)	16.9% (*n* = 15)	41.6% (*n* = 37)	21.3% (*n* = 19)	15.7% (*n* = 14)
Non-binary AMAB	2.1% (*n* = 1)	10.6% (*n* = 5)	59.6% (*n* = 28)	17.0% (*n* = 8)	10.6% (*n* = 5)
Control males [31]	1.4% (*n* = 10)	5.8% (*n* = 42)	45.9% (*n* = 332)	24.1% (*n* = 174)	22.8% (*n* = 165)

Extreme Type E = Much stronger empathising relative to systemising; Type E = Stronger empathising relative to systemising; Type B = Balanced; similar levels of systemising and empathising; Type S = Stronger systemising relative to empathising; Extreme Type S = Much stronger systemising relative to empathising

### Supplementary analyses

For completeness, correlations between the measures were examined. Potential group differences in mean scores on the measures between transgender men and non-binary AFAB and between transgender women and non-binary AMAB were also explored. These findings are reported in the Additional file 1.

## Discussion

In the present study, all the comparisons between gender minority adults assigned female at birth and control females yielded significant results in the expected directions in line with the EMB [12] and the mindblindness theory [25]. Gender minority adults assigned female at birth scored higher on the AQ and the SQ-Short but lower on the EQ-Short, the EQ-10, and the Eyes Test. These current findings suggest that, on average, transgender men and non-binary AFAB show a “masculinised” autism-related profile and reduced ToM. The finding that autistic traits are elevated in transgender men and non-binary AFAB concurs with findings from most prior studies [1–5].

As for comparisons between gender minority adults assigned male at birth and control males, there were no differences in autistic trait and findings regarding the other measures were mixed. Due to a lack of an increase in autistic traits in transgender women or non-binary AMAB in the present study, it is hard to interpret the relevant findings within the context of the EMB or the mindblindness theory. These theories are typically used to explain the development of autism or elevated autistic traits. It is unclear how one may apply these theories when a study population does not show elevated autistic traits. However, the current finding suggesting no difference in autistic traits between gender minority adults assigned male at birth and control males is consistent with findings from some prior studies [1, 2, 6].

Heightened early androgen exposure may play a role in shaping the “masculinised” autism-related profile observed in gender minority adults assigned female at birth. Nonetheless, the behavioural effects of early androgen exposure on human gender development have been documented in both males and females [23, 24]. It is unclear why there was a sex-specific pattern in the present study; gender minorities assigned female at birth showed a more “masculine” autism-related profile, but gender minorities assigned male at birth did not show a more “feminine” autism-related profile. Noteworthy is that the hypotheses related to the EMB and the mindblindness theory were not developed to explain autistic traits in gender minorities. It would be useful for further research to extend the existing theoretical frameworks to formulate more specific theories that address autism-related issues in gender minorities.

The present study suggests that some gender minority adults, especially those assigned female at birth, might have rigid thinking styles and might experience socio-cognitive difficulties. Some gender minority adults may seek clinical and other professional services in different settings. Professionals working in various settings may need to attend to the relevant issues and adjust their communication styles when they provide support to gender minority adults.

## Limitations

The AQ is not a diagnostic tool, and so high-scoring individuals may not necessarily receive a clinical diagnosis of autism. It has also been argued that the measures employed in the present study may not provide a precise assessment of the theoretical constructs of interest [39–41]. However, different versions of the employed measures are commonly used in studies testing the EMB and the mindblindness theory. There is also an increasing amount of research examining SQ and EQ scores in gender minority adults [11, 42]. Using these measures may enable comparisons across different study populations. In particular, a recent large-scale general population study found increased autistic traits and systemising but reduced empathising in gender minority individuals compared with cisgender individuals, although it was not possible for the study to make comparisons based on sex assigned at birth due to the way the sex/gender question was asked in the study [11].

The present study attempted to make comparisons to different control groups to see if the effects can be replicated across samples. There were two control samples for the AQ [34, 35] and the EQ measures [31, 32]. However, for the Eyes Test, there has only been one large-scale general populations study [36]. The largest study measuring systemising and empathising employed the EQ-10 and the SQ-10 [32]. Whilst the EQ-10 can be derived using items on the EQ-Short, the SQ-10 cannot be derived using items on the SQ-Short. Hence, there was only one control sample for the SQ measure [31].

The present study relied on previously published descriptive statistics from various studies to make statistical comparisons, and thus it was difficult to adopt a conventional approach that would involve testing interactions before examining subgroup differences. However, interaction terms in statistical models may not detect meaningful or subtle patterns in studies without enormous subgroup samples. More importantly, there seems to be a clear pattern in the data suggesting a “masculinised” autism-related profile in gender minority adults assigned female at birth and generally unaltered scores on the relevant measures in gender minority adults assigned male at birth. In particular, for the comparisons between gender minority adults assigned female at birth and control females, all the effects concerning AQ, SQ, and EQ scores were moderate to large (Cohen’s *d* = 0.47 to 1.24) and highly significant (all *p* values < 0.001). Although the present study did not apply Bonferroni correction to individual *p* values, these specific results would have remained significant after correction for multiple comparisons. The effects concerning ToM in gender minority adults assigned female at birth were smaller (Cohen’s *d* = 0.32 to 0.36) and less significant (*p* = 0.008 to 0.025). Like many other studies focusing on minority groups, the present study does not have a large sample. Further research is needed to corroborate the current findings, especially those on ToM. Further research may continue to investigate the mechanisms shaping autistic traits in both transgender and non-binary individuals.

The current sample was not recruited through a clinic. Nonetheless, not all gender minority adults have access to or require clinical services. Hence, compared with samples recruited through clinics, the current sample may be more representative of the gender minority community as a whole.

## Conclusions

The current findings suggest that, on average, transgender men and non-binary AFAB show a “masculinised” autism-related profile and reduced ToM. These findings might be interpreted to support the EMB and the mindblindness theory. Further research is needed to corroborate the current findings.

## Supplementary information

**Additional file 1.** Supplementary analyses.

## Data Availability

The dataset generated and analysed during the current study is available from the corresponding author on reasonable request.

## Abbreviations

AFAB: Individuals assigned female at birth
AMAB: Individuals assigned male at birth
AQ: Autism Spectrum Quotient
EMB: Extreme male brain theory of autism
EQ-Short: Short version of the Empathy Quotient
EQ-10: 10-Item version of the Empathy Quotient
Eyes Test: Revised version of the Reading the Mind in the Eyes Test
SQ-Short: Short version of the Systemising Quotient
ToM: Theory of Mind
