# Supplementary material for: Autistic traits, systemising, empathising, and theory of mind in transgender and non-binary adults
Source: Mol Autism. 2020 Sep 29;11:73. doi: 10.1186/s13229-020-00378-7 (PMC7523342; doi:10.1186/s13229-020-00378-7)
Supplement: Supplementary file 1 — Additional file 1. Supplementary analyses. [file 13229_2020_378_MOESM1_ESM.pdf]

ADDITIONAL FILE
Supplementary Analyses

Correlations between Measures

Pearson's correlations were conducted to examine the relations between the measures in the current sample and the findings are summarised in Table S1. The AQ significantly correlated with each of the other measures. There were no significant correlations between EQ and SQ scores or between the Eyes Test and the EQ/SQ measures. The EQ-Short and the EQ-10 significantly strongly correlated with each other.

Table S1. Correlations between measures.

	AQ	SQ-Short	EQ-Short	EQ-10	Eyes Test
AQ	-	-	-	-	-
SQ-Short	$r = .18$<br>$p = .002$	-	-	-	-
EQ-Short	$r = -.71$<br>$p < .001$	$r = -.08$<br>$p = .16$	-	-	-
EQ-10	$r = -.67$<br>$p < .001$	$r = -.07$<br>$p = .24$	$r = .95$<br>$p < .001$	-	-
Eyes Test	$r = -.13$<br>$p = .02$	$r = -.07$<br>$p = .23$	$r = .09$<br>$p = .10$	$r = .08$<br>$p = .17$	-

Comparisons between Transgender and Non-Binary Individuals

Group differences in mean scores on the various measures between transgender men and non-binary AFAB and between transgender women and non-binary AMAB were evaluated using the Cohen's *d* statistic and independent samples *t*-tests. The effect sizes and inferential statistics are summarised in Table S2 in this document. Descriptive statistics for the subgroups are summarised in Table 1 in the main text. There was only one significant group difference; non-binary AFAB scored higher on the AQ than did transgender men. All other group comparisons yielded non-significant results and small to negligible effects. No statistical tests were conducted to compare the proportion of high-scoring or the distribution of "brain types" due to the small samples in some of the individual cells (see main text for descriptive statistics). However, the relevant descriptive statistics seem to suggest that there were little to no differences in high-scoring or "brain types" between transgender men and non-binary AFAB or between transgender women and non-binary AMAB. Taken together,

these findings suggest that transgender and non-binary individuals are largely similar in terms of EMB-related traits and ToM.

Table S2. Inferential statistics and effect sizes for the comparisons between transgender and non-binary individuals regarding mean scores.

Comparison	Measure	*t*	*p*	*d*[1]
Transgender Men v.s. Non-Binary AFAB	AQ	2.65	.01	0.41
	SQ-Short	0.30	.77	0.05
	EQ-Short	-0.36	.72	-0.06
	EQ-10	-0.05	.96	-0.01
	Eyes Test	0.24	.81	0.04
Transgender Women v.s. Non-Binary AMAB	AQ	1.21	.23	0.22
	SQ-Short	-0.44	.66	-0.08
	EQ-Short	-0.43	.67	-0.08
	EQ-10	-0.25	.81	0.04
	Eyes Test	1.06	.29	0.19

[1]Positive *d* values indicate higher scores in non-binary individuals.
